# Management of obstetric and gynecologic hemorrhage: a narrative review of current guidelines and evidence

**DOI:** 10.25122/jml-2026-0036

**Published:** 2026-03

**Authors:** Mihaela Ichim, Alina-Gabriela Marin, Mihaela Bot, Andreea Borislavschi, Andreea Gratiana Boiangiu, Radu Vladareanu, Alexandru Filipescu

**Affiliations:** 1 Department of Obstetrics and Gynecology, Elias University Emergency Hospital, Carol Davila University of Medicine and Pharmacy, Bucharest, Romania

**Keywords:** obstetric hemorrhage, postpartum hemorrhage, gynecologic bleeding, abnormal uterine bleeding, uterotonics, tranexamic acid, clinical guidelines

## Abstract

Obstetric hemorrhage remains one of the leading causes of maternal morbidity and mortality worldwide despite clinical guidelines and effective interventions. Postpartum hemorrhage is the most common and clinically significant form, yet variability persists across guidelines regarding diagnostic criteria, blood-loss assessment, transfusion thresholds, and escalation strategies. In parallel, gynecologic hemorrhage, particularly acute abnormal uterine bleeding, remains an important cause of morbidity and requires a distinct therapeutic approach. This study was conducted as a structured narrative review informed by systematic search principles. A literature search was performed in PubMed, Scopus, and Web of Science for studies published between January 2020 and January 2026, supplemented by targeted searches of guideline repositories and official organizational websites. The review focused on international guidelines and relevant studies on diagnosis, treatment, transfusion practices, and escalation pathways for obstetric and gynecologic hemorrhage. Of 1,248 records identified, 32 studies were included in the final synthesis. Strong convergence was observed regarding early recognition and administration of uterotonics, particularly oxytocin, as first-line therapy, with early use of tranexamic acid as an adjunct within three hours of birth. Increasing evidence supports protocol-driven care over traditional stepwise escalation. Restrictive transfusion strategies are recommended for stable patients, while clinical status remains the primary determinant in patients with active bleeding. Acute abnormal uterine bleeding management relies on antifibrinolytic and hormonal therapy. Contemporary hemorrhage management is increasingly early, physiology-based, multidisciplinary, and protocolized. Optimal outcomes depend on rapid recognition, prompt first-line treatment, and timely escalation. Integrating principles from gynecologic hemorrhage may further strengthen individualized and etiology-driven care.

## Introduction

Obstetric hemorrhage remains a leading cause of maternal morbidity and mortality worldwide, accounting for approximately one quarter of maternal deaths globally [1,2]. Recent estimates suggest that postpartum hemorrhage (PPH) complicates approximately 5–10% of all deliveries, with higher rates reported in low-resource settings [3-5]. PPH is the most frequent form of severe obstetric bleeding and has traditionally been defined as blood loss exceeding 500 mL after vaginal birth or 1000 mL after cesarean delivery [6-8]. However, contemporary evidence increasingly challenges the exclusive use of volume-based thresholds, as clinical deterioration may occur before visual estimates or laboratory changes fully reflect the severity of blood loss [4,6-8].

Over the past decade, major international organizations, including the World Health Organization (WHO), the International Federation of Gynecology and Obstetrics (FIGO), the American College of Obstetricians and Gynecologists (ACOG), and the Royal College of Obstetricians and Gynecologists (RCOG), have progressively refined their recommendations to encourage earlier recognition, faster treatment, and a more structured multidisciplinary response [3,4,7,8]. These developments have paralleled advances in patient blood management, a better understanding of hemorrhage pathophysiology, and stronger evidence supporting interventions such as tranexamic acid (TXA) and care-bundle strategies [9-20].

In recent years, the management paradigm has shifted from sequential escalation toward earlier, combined intervention. Current frameworks increasingly support the simultaneous initiation of key measures, including uterotonics, tranexamic acid, intravenous access, fluid resuscitation, and etiologic assessment, rather than delaying escalation until individual steps have failed [3,12,17-20]. Nevertheless, uterotonics, particularly oxytocin, remain the cornerstone of first-line therapy and should be administered immediately following placental delivery as part of active management of the third stage of labor. Despite this broad convergence, heterogeneity persists across international guidelines, particularly regarding diagnostic thresholds, objective blood-loss measurement, transfusion triggers, and criteria for escalation to advanced interventions such as tamponade, interventional radiology, or surgery [3,7,8,18-23].

Advanced surgical options also play a critical role in refractory cases, particularly uterus-sparing techniques such as uterine compression sutures and internal iliac artery ligation, which may provide life-saving hemorrhage control while preserving future reproductive potential [24-28].

The aim of this study was to provide a structured narrative review of major international guidelines and recent supporting literature, with particular emphasis on clinically relevant management thresholds and practical case-management strategies in obstetric hemorrhage. In routine clinical practice, delays in recognition and escalation remain among the most important contributors to adverse outcomes.

Although obstetric hemorrhage represents the main focus of this review, gynecologic hemorrhage, particularly abnormal uterine bleeding (AUB), remains an important clinical entity associated with chronic morbidity, impaired quality of life, and increased healthcare utilization. Contemporary approaches emphasize structured etiologic classification and individualized management strategies based on the FIGO Systems 1 and 2 framework and the PALM-COEIN classification [29,30]. In gynecologic practice, acute AUB may also present as a hemorrhagic emergency requiring rapid stabilization and targeted therapy. Unlike postpartum hemorrhage, its management relies more heavily on pharmacologic hemostasis, including antifibrinolytics, high-dose hormonal therapy, and correction of underlying coagulopathies [31-33].

## Material and Methods

This study was designed as a structured narrative review informed by systematic search principles. The review focused on international guideline documents issued by major authoritative organizations, including WHO, FIGO, ACOG, and RCOG, as well as recent peer-reviewed studies that contextualized and evaluated key recommendations in contemporary clinical practice.

A structured literature search was conducted in PubMed, Scopus, and Web of Science for articles published between January 2020 and January 2026. Additional targeted searches were performed in guideline repositories and on organizational websites to identify the most recent official guidance documents. Search terms included combinations of “postpartum hemorrhage,” “obstetric hemorrhage,” “gynecologic bleeding,” “tranexamic acid,” “uterotonics,” “blood transfusion threshold,” “massive transfusion protocol,” “objective blood loss measurement,” and “clinical guidelines.” Particular attention was also given to studies addressing abnormal uterine bleeding and gynecologic hemorrhage to provide a complementary perspective to obstetric bleeding management.

Although the review followed a structured search strategy inspired by the PRISMA methodology, it was designed as a narrative synthesis of guideline-based and clinically relevant evidence, without a formal risk-of-bias assessment or a quantitative meta-analysis. Consensus statements and implementation studies were also included when they provided clinically relevant insights into real-world management and guideline application.

### Eligibility criteria

Studies were considered eligible if they were randomized controlled trials, systematic reviews, meta-analyses, guideline documents, consensus statements, or implementation studies relevant to the prevention, diagnosis, or treatment of obstetric or gynecologic hemorrhage and if they included clinically applicable management data, such as the timing of TXA administration, escalation pathways, or transfusion thresholds.

Studies were excluded if they were case reports or small descriptive case series, were published in languages other than English, lacked clear clinical applicability, or represented duplicate publications or superseded versions of the same guidance.

### Study selection

**Figure 1:**
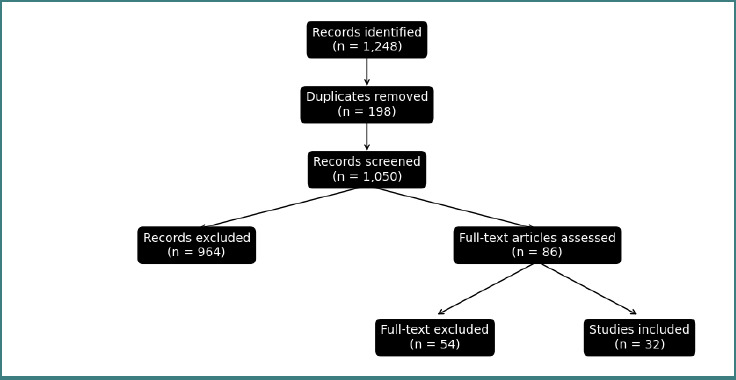
Flow diagram of study selection process. A total of 1,248 records were identified through database searching. After removal of duplicates, 1,050 records were screened. Eighty-six full-text articles were assessed for eligibility, and 32 studies were included in the final analysis.

A total of 1,248 records were identified. After duplicate removal, 1,050 records underwent title and abstract screening. Eighty-six full-text articles were assessed for eligibility, and 32 studies were included in the final synthesis (Figure 1). Studies excluded at the full-text stage were removed because they did not report clinically relevant management thresholds, were not guideline-based, were outdated in relation to current practice, focused on non-obstetric populations, or had limited methodological quality.

### Data extraction and synthesis

The analysis focused on predefined domains, including prevention strategies, diagnostic criteria, and early recognition, pharmacological management, non-pharmacological and procedural interventions, transfusion thresholds and blood-component therapy, and escalation pathways with system-level implementation.

Because the primary objective was to synthesize and compare recommendations rather than generate pooled quantitative estimates, no formal meta-analysis was performed. Instead, a narrative comparative approach was used to identify areas of convergence, divergence, and practical clinical implications.

## Results

### Study selection and included evidence

The final synthesis included 32 publications, comprising major international guideline documents, randomized controlled trials, systematic reviews, meta-analyses, consensus statements, and implementation studies. The evidence base was dominated by publications from the last 5 years, although a limited number of older, yet highly influential, guideline documents were retained due to their ongoing clinical relevance. Table 1 summarizes the major international guidelines included in this review and their dominant management recommendations.

**Table 1:** Summary of major international guidelines on postpartum hemorrhage

Guideline	Year	First-line therapy	TXA recommendation	Escalation strategy	Key approach
WHO	2025	Oxytocin (first-line uterotonic)	Within 3 hours (1 g IV, repeat if needed)	Early bundled escalation	Physiology-based, bundled care
FIGO	2022	Oxytocin + active management of the third stage	Early administration recommended	Simplified algorithm	Rapid response, low-resource adaptability
ACOG	2017	Uterotonics (oxytocin first-line)	Recommended as an adjunct	Stepwise escalation	Structured escalation
RCOG	2017	Uterotonics (oxytocin first-line)	Included in management protocols	Protocol-based escalation	Multidisciplinary approach

### Consensus across guidelines

Across all major international guideline frameworks, several core principles of hemorrhage management were consistently emphasized. Active management of the third stage of labor and routine use of uterotonic agents, particularly oxytocin, remain the foundation of prevention and first-line treatment [3,4,7,8,15,16,34]. Early recognition of hemorrhage is universally endorsed, although newer WHO and FIGO recommendations place greater emphasis on physiological instability and objective blood-loss assessment rather than relying on visual estimation alone [3,4,21,35].

TXA has emerged as a central adjunct in contemporary management. International guidance strongly supports its early administration, ideally as soon as possible and within 3 hours of birth, based on trial evidence demonstrating a reduction in death due to bleeding [12-14]. Guidelines also consistently endorse multidisciplinary team activation, rapid access to blood products, and standardized escalation pathways.

Although gynecologic bleeding is less extensively standardized than postpartum hemorrhage, its management similarly relies on etiologic classification and targeted therapy, particularly in cases of acute abnormal uterine bleeding.

### Differences between guidelines

Despite broad agreement, important differences remain. One major distinction concerns how PPH is defined and recognized. WHO guidance increasingly prioritizes clinical indicators and objective blood-loss measurement, whereas RCOG and ACOG retain stronger links to conventional quantitative thresholds combined with clinical assessment [3,7,8,21]. Another area of divergence is treatment sequencing. While older frameworks describe a more stepwise progression from uterotonics to tamponade and then surgery, WHO- and FIGO-influenced models increasingly favor bundled approaches in which multiple interventions are initiated simultaneously [3,4,9-11]. Table 2 highlights the dominant management orientation of the major guideline frameworks.

**Table 2:** Comparative overview of guideline orientation in PPH management

Guideline	Year	Recognition approach	Management model	TXA timing	Transfusion strategy
WHO	2025	Physiological + objective blood loss	Bundled care	≤3 hours	Early if instability
FIGO	2022	Early clinical recognition	Algorithm-based	Early use	Context-dependent
ACOG	2017	Volume + clinical signs	Stepwise	Adjunctive	Restrictive threshold
RCOG	2017	Combined criteria	Protocol-driven	Included	Guided by clinical status

### Clinically relevant management thresholds

Several practical management thresholds emerged from the literature. TXA is recommended at a dose of 1 g intravenously, administered as early as possible and within 3 hours, with a second 1 g IV dose if bleeding continues after 30 minutes or restarts within 24 hours [12-14]. For blood-component therapy, a restrictive transfusion approach is generally supported in stable patients, with hemoglobin <7 g/dL considered a reasonable threshold for transfusion in the absence of active instability [17-20]. However, in acute obstetric hemorrhage, transfusion should be considered earlier when there is ongoing bleeding, tachycardia, hypotension, symptomatic anemia, impaired tissue perfusion, or limited physiologic reserve.

In addition to tranexamic acid, uterotonic regimens remain central to first-line management and should be administered using standardized dosing protocols. Oxytocin is typically given as 10 IU intravenously or intramuscularly, followed by continuous infusion (10–40 IU diluted in 500–1000 mL crystalloid). If bleeding persists, second-line uterotonics include methylergometrine (0.2 mg IM/IV, repeated every 2–4 hours; contraindicated in hypertension), carboprost (250 µg IM, repeated every 15–90 minutes up to a maximum cumulative dose of 2 mg; contraindicated in asthma), and misoprostol (800–1000 µg administered sublingually or rectally) [3,6,15,16,34].

Fibrinogen replacement should be considered when levels fall below 2 g/L, as hypofibrinogenemia is an early marker of severe hemorrhage. Where available, viscoelastic testing may support targeted coagulation therapy and help optimize transfusion strategies [18-20,36].

**Figure 2:**
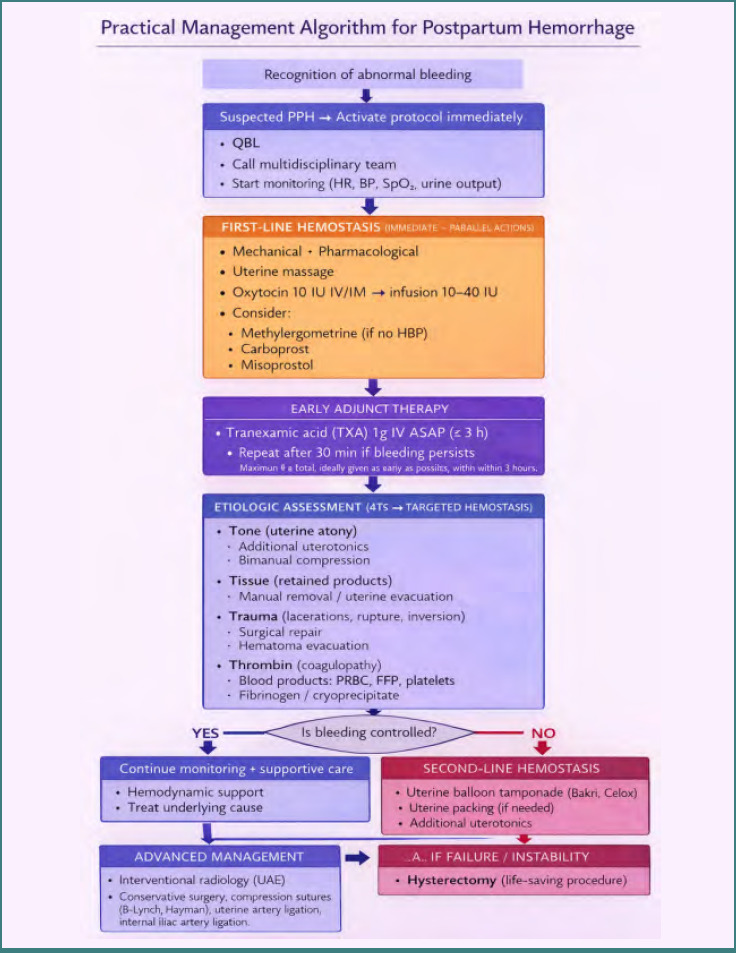
**Stepwise management algorithm for postpartum hemorrhage, from early recognition and resuscitation to targeted etiologic treatment and escalation to second-line, advanced, and definitive hemostatic interventions in cases of persistent bleeding or hemodynamic instability**. 4Ts, Tone, Tissue, Trauma, Thrombin; ASAP, as soon as possible; FFP, fresh frozen plasma; HBP, high blood pressure; HR, heart rate; IM, intramuscular; IU, international units; IV, intravenous; PPH, postpartum hemorrhage; PRBC, packed red blood cells; QBL, quantitative blood loss; SpO_2_, peripheral oxygen saturation; TXA, tranexamic acid; UAE, uterine artery embolization.

Clinical decision-making should remain dynamic and guided by ongoing bleeding and hemodynamic status rather than by isolated numerical values. A structured, physiology-based management approach integrating these elements is summarized in Figure 2.

Massive transfusion protocols are typically activated in cases such as blood loss >1500 mL, anticipated need for rapid multi-unit transfusion, or persistent uncontrolled bleeding, and commonly use component ratios approximating 1:1:1 for red blood cells, plasma, and platelets [18-20].

Second-line interventions include uterine tamponade, vacuum-induced hemorrhage-control devices, and uterine-sparing surgical procedures such as uterine compression sutures and internal iliac artery ligation in selected refractory cases [24,37,38]. In addition to these techniques, internal iliac artery ligation (IIAL) remains an important uterus-sparing surgical option in the management of severe and refractory obstetric hemorrhage [24-27]. By significantly reducing pelvic arterial pressure and pulse pressure distal to the ligation site, IIAL facilitates effective hemostasis while maintaining sufficient collateral circulation to prevent tissue necrosis [25]. Clinical studies and cumulative surgical experience have shown that IIAL can be a life-saving intervention, particularly in cases of uterine atony, placenta accreta spectrum disorders, or pelvic trauma when pharmacologic and minimally invasive measures fail [25-27].

Although the procedure is technically demanding and requires adequate surgical expertise, it offers a valuable alternative to emergency hysterectomy, especially in women desiring future fertility [26,28]. Importantly, IIAL remains highly relevant in settings where interventional radiology is unavailable or delayed, further supporting its inclusion in comprehensive escalation algorithms for severe obstetric hemorrhage [26,27].

These strategies are increasingly incorporated into practical escalation algorithms designed to control bleeding while preserving reproductive potential whenever feasible.

### System-level findings

Implementation studies and care-bundle reports showed that protocolized response, simulation training, hemorrhage carts, standardized checklists, and improved communication with transfusion services reduce delays and improve adherence to guidelines [9-11,39]. These findings were especially consistent across organized maternity systems and support the view that maternal outcomes depend not only on what is recommended but also on how reliably those recommendations are implemented. Recent reports also emphasize that implementation in low-resource settings requires adaptation to local availability of blood products, uterotonics, trained personnel, and referral systems [40,41].

These findings provide the basis for the clinical interpretation developed in the Discussion section.

## Discussion

Obstetric hemorrhage remains a leading cause of maternal morbidity and mortality worldwide, and recent WHO-linked cause-of-death analyses confirm that hemorrhage continues to be the leading direct cause of maternal death globally [1,2]. In clinical practice, postpartum hemorrhage remains the dominant phenotype of severe obstetric bleeding, but the contemporary literature increasingly challenges a purely volume-based definition. Traditional thresholds of more than 500 mL after vaginal birth and more than 1000 mL after cesarean delivery remain useful for communication and surveillance, yet they may be insufficient for bedside decision-making because acute clinical deterioration can occur before estimated blood-loss thresholds or laboratory abnormalities become fully apparent [6-8]. This tension between numerical definitions and physiology-based recognition is one of the central themes emerging from the present review.

A key finding of this analysis is the clear international convergence toward earlier identification and faster treatment. The 2025 WHO consolidated guideline emphasizes prevention, diagnosis, and treatment within a unified PPH framework, while the 2022 FIGO recommendations similarly move toward simplified, actionable management algorithms [3,4]. Although the ACOG Practice Bulletin and the RCOG Green-top Guideline remain foundational references, their operationalization increasingly overlaps with newer WHO- and FIGO-aligned concepts, particularly regarding rapid team activation, protocol-based responses, and minimizing delays [7,8]. This evolution reflects a broader recognition that PPH is not a static event but a rapidly evolving syndrome in which uterine atony, soft tissue trauma, retained tissue, and coagulopathy may coexist and amplify one another.

Another important development is the transition from classic sequential escalation toward bundled care. Historically, management was often described as a stepwise ladder: uterine massage and oxytocin first, followed by additional uterotonics, then tamponade or surgery if bleeding persisted [7,8]. However, the WHO technical consultation on PPH care bundles and the E-MOTIVE trial shifted attention toward simultaneous early intervention [9,10]. In E-MOTIVE, objective blood-loss measurement combined with a treatment bundle significantly reduced severe PPH-related outcomes compared with usual care [10]. A 2024 systematic review further supported the effectiveness of care bundles for the prevention and treatment of PPH, although heterogeneity in implementation remains substantial [11]. Clinically, this suggests that waiting for one step to fail before initiating the next may no longer represent best practice in many settings. At the same time, the timely initiation of simple first-line measures, particularly oxytocin administration and uterine massage, remains essential and should not be overshadowed by emphasis on adjunctive therapies.

The role of TXA is one of the strongest areas of consensus across international guidance. The WOMAN trial demonstrated that TXA reduces death due to bleeding when administered early in women with PPH [12]. Subsequent analyses and guideline updates reinforced the importance of the treatment window, supporting administration as soon as possible and within 3 hours of birth, with a standard dose of 1 g IV and a second 1 g dose if bleeding persists for 30 minutes or recurs within 24 hours [13,14]. This timing is clinically important because delayed recognition is one of the main reasons why effective therapies lose part of their benefit. Accordingly, TXA should be integrated into the initial response algorithm rather than reserved for late rescue use.

The review also confirms that uterotonics remain the pharmacologic cornerstone of both prevention and early treatment. Oxytocin remains the preferred first-line uterotonic in most guidelines, whereas other options, such as misoprostol, ergometrine-based regimens, and carbetocin, have more context-dependent roles [3,4,15,16,34]. Network meta-analysis data indicate that several uterotonic agents are effective in preventing PPH, but their relative ranking depends on the outcome assessed and on adverse-effect profiles [15]. This is clinically relevant because protocol design must account not only for efficacy, but also for drug availability, cold-chain reliability, route of administration, and tolerability, particularly in low-resource settings [16].

With regard to transfusion and blood-component therapy, the available evidence supports a restrictive transfusion philosophy in stable patients while preserving clinician flexibility in active hemorrhage [17-19]. In the broader transfusion literature, restrictive thresholds around hemoglobin 7–8 g/dL are generally supported [17]. In obstetric hemorrhage, however, laboratory hemoglobin may lag behind the true severity of blood loss, especially in acute settings before redistribution has occurred [18,19]. For that reason, most contemporary frameworks recommend transfusion at Hb <7 g/dL in stable patients, but earlier transfusion should be considered when there is ongoing bleeding, hemodynamic instability, symptomatic anemia, poor physiologic reserve, or evidence of inadequate oxygen delivery [18-20]. Massive transfusion protocols are typically activated in cases such as persistent uncontrolled bleeding, blood loss >1500 mL, or the anticipated need for rapid transfusion of multiple red-cell units, and are commonly organized around balanced component therapy approximating a 1:1:1 ratio of red blood cells, plasma, and platelets [18-20]. In practice, transfusion decisions in obstetrics are best understood as physiology-led rather than hemoglobin-led.

Another important theme is the growing emphasis on objective blood-loss assessment. Visual estimation remains widely used, but it is repeatedly shown to underestimate actual blood loss, especially as severity increases [10,21,35]. Newer WHO and FIGO guidance supports more objective quantification methods where feasible, and FIGO’s 2025 recommendations specifically address objective blood-loss measurement after birth for early detection of PPH [21]. Nevertheless, implementation varies considerably. High-resource centers may be more likely to adopt calibrated drapes and standardized quantitative blood-loss protocols, whereas lower-resource settings may rely on simplified measurement tools and structured clinical observation. Accurate measurement should therefore be encouraged, but it should not delay treatment when clinical instability is already evident.

From an etiologic perspective, the findings of this review continue to support the classic “4Ts” framework—tone, tissue, trauma, and thrombin—as a practical and pathophysiologically coherent way to structure assessment [5,6,18]. Recent large-scale meta-analyses confirm that uterine atony remains the leading cause of PPH, while antenatal anemia, placental abnormalities, hypertensive disease, obesity, and other modifiable or identifiable risk factors influence severity and outcomes [5,22]. The association between prepartum anemia and subsequent PPH is especially important because it links antenatal care to intrapartum resilience: women who enter labor with depleted iron stores or anemia may tolerate blood loss poorly and may require transfusion earlier in the course of hemorrhage [22,23]. This supports a broader patient blood management approach extending beyond the delivery room.

The literature also highlights the importance of implementation science and safety bundles. Evidence from quality-improvement initiatives and hospital-level bundle implementation studies shows that standardized pathways, simulation-based training, hemorrhage carts, rapid blood-bank communication, and team rehearsal can reduce treatment delays and improve outcomes [9-11,39]. In routine clinical practice, delays in recognition and escalation remain among the most important contributors to adverse outcomes. This persistent gap between guideline recommendations and bedside practice suggests that outcomes may depend less on what a guideline recommends than on how reliably teams execute those recommendations in a timely manner.

The evidence base has also expanded regarding second-line and fertility-preserving interventions. Intrauterine balloon tamponade and vacuum-induced hemorrhage-control devices are increasingly used in cases of persistent bleeding after initial pharmacologic therapy and may reduce the need for hysterectomy in selected patients [37,38]. When conservative methods fail, uterine-sparing surgical procedures remain important options before definitive hysterectomy, especially in women who wish to preserve fertility [24]. These escalation strategies reinforce the importance of a timely transition from first-line to second-line care rather than prolonged ineffective pharmacologic treatment.

Although the strongest convergence across international guidelines is observed in postpartum hemorrhage, the broader framework of hemorrhage management in obstetrics and gynecology also benefits from structured etiologic classification and targeted intervention. In gynecologic practice, abnormal uterine bleeding represents a major cause of morbidity and healthcare utilization, but differs fundamentally from obstetric hemorrhage in both pathophysiology and management. FIGO-based systems emphasize structured etiologic classification using the PALM-COEIN framework, allowing more precise diagnosis and individualized treatment strategies [29,30].

**Figure 3:**
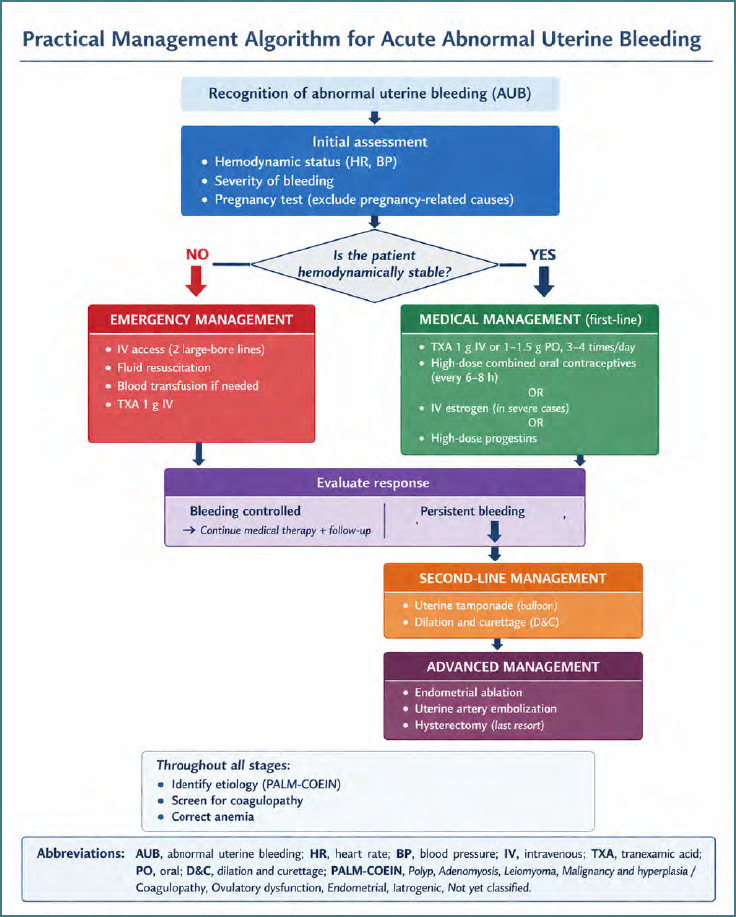
**Practical management algorithm for acute abnormal uterine bleeding (AUB). This algorithm outlines a structured, etiology-based approach to the evaluation and treatment of acute AUB. Initial steps include assessment of hemodynamic stability and exclusion of pregnancy**. Hemodynamically unstable patients require emergency resuscitation, early administration of tranexamic acid (TXA), and transfusion if needed. Stable patients are managed with medical therapy, including TXA and high-dose hormonal regimens. If bleeding persists, second-line procedures such as uterine tamponade or dilation and curettage (D&C) are considered, followed by advanced interventions in refractory cases. Etiologic classification using the PALM-COEIN system, screening for coagulopathy, and correction of anemia should be integrated throughout all stages.

Acute abnormal uterine bleeding is typically managed with pharmacologic hemostasis rather than uterotonic therapy, including tranexamic acid, high-dose estrogen or combined hormonal regimens, and progestins. In selected refractory cases, procedural options such as intrauterine tamponade, dilation and curettage, or endometrial ablation may be required [31-33]. To better illustrate the differences between obstetric and gynecologic hemorrhage management, a structured algorithm for the management of acute abnormal uterine bleeding is presented in Figure 3.

In addition to this algorithmic approach, key conceptual differences between obstetric and gynecologic hemorrhage management are summarized in Table 3.

**Table 3:** Comparative overview of obstetric and gynecologic hemorrhage management

Element	Obstetric hemorrhage (PPH)	Gynecologic hemorrhage (AUB)
Main cause	Uterine atony	Hormonal or structural imbalance
First-line therapy	Uterotonics	TXA and hormonal therapy
Clinical setting	Acute postpartum	Acute or chronic
Classification system	4Ts (Tone, Tissue, Trauma, Thrombin)	PALM-COEIN

This table summarizes the key differences between obstetric hemorrhage (postpartum hemorrhage, PPH) and gynecologic hemorrhage (acute abnormal uterine bleeding, AUB) with respect to underlying etiology, first-line therapeutic approach, clinical context, and classification systems. While PPH is typically driven by uterine atony and managed primarily with uterotonics within an acute postpartum setting, AUB is more commonly associated with hormonal or structural causes and is managed predominantly with antifibrinolytic and hormonal therapies. These distinctions highlight the importance of adapting management strategies to the underlying pathophysiology.

Despite these differences, a common principle remains evident across obstetrics and gynecology: optimal hemorrhage management depends on early recognition, etiologic assessment, and timely initiation of targeted therapy.

Taken together, the literature supports a practical contemporary management model: prompt recognition of abnormal bleeding; immediate first-response treatment including uterotonics, TXA, intravenous access, and resuscitation; rapid etiologic reassessment using the 4Ts framework; early escalation to tamponade or surgery when bleeding persists; and judicious but timely use of blood components and massive transfusion protocols when clinically indicated [3,4,12-20,24,37,38]. The overall direction of international guidance is therefore clear. Modern hemorrhage care is becoming faster, more protocolized, more physiology-based, and more multidisciplinary. The major challenge is no longer the absence of effective interventions, but the consistent and timely implementation of these interventions across diverse healthcare systems.

### Clinical management synthesis

Based on the available evidence, contemporary management of obstetric hemorrhage should follow a structured, physiology-based approach that prioritizes early recognition, immediate initiation of uterotonics and tranexamic acid, rapid hemodynamic stabilization, and concurrent etiologic assessment using the 4Ts framework. Oxytocin should be initiated immediately after placental separation as first-line therapy, typically as 10 IU IV/IM followed by infusion, while TXA should be administered as 1 g IV as early as possible and within 3 hours of birth, with repeat dosing when indicated [12-16,34].

Escalation to second-line uterotonics, uterine tamponade, vacuum-induced hemorrhage-control systems, interventional radiology, or advanced surgical procedures should occur without delay when bleeding persists [24,34,37,38]. Blood transfusion should generally be considered in stable patients with hemoglobin <7 g/dL, but decisions in acute hemorrhage should primarily be guided by clinical status, ongoing blood loss, and tissue perfusion rather than hemoglobin alone [17-20,36]. This integrated approach reflects the transition toward protocol-driven, multidisciplinary care aimed at minimizing delays and improving outcomes.

### Limitations

This review has several limitations. First, although it followed a structured search strategy, the included literature comprised both formal guidelines and heterogeneous secondary evidence, limiting direct comparability across sources. Second, some key obstetric guidelines that are older than 5 years remain highly influential and were included for completeness, even though recent evidence was prioritized. Third, several implementation studies and quality-improvement reports used different outcome definitions, bundle components, and thresholds for escalation, making the synthesis more interpretive than quantitative. Fourth, transfusion thresholds in obstetrics cannot be extrapolated directly from general adult transfusion studies, so some recommendations necessarily depend on expert consensus and clinical judgment rather than obstetric-specific randomized evidence. Finally, most high-quality implementation data come from better-resourced systems, which may limit generalizability to low-resource settings where delays, shortages of blood products, and workforce constraints are more pronounced [3,4,11,18,39-41].

## Conclusion

Obstetric hemorrhage remains a major and still partly preventable cause of severe maternal morbidity and mortality worldwide. The evidence synthesized in this review shows broad agreement across major international guidelines on several core principles: early recognition, uterotonics as first-line therapy, rapid administration of tranexamic acid within 3 hours, multidisciplinary response, and timely escalation when bleeding persists. Contemporary management is increasingly shaped by bundled-care models and objective blood-loss assessment, while transfusion practice is moving toward restrictive hemoglobin thresholds in stable patients but remains fundamentally guided by clinical status in acute hemorrhage.

In practical terms, the most robust case-management strategy is an early, protocol-driven, physiology-based approach that combines immediate first-response measures with structured reassessment and timely escalation. Future progress will depend less on discovering entirely new treatments and more on harmonizing guidance, improving implementation, and ensuring equitable access to evidence-based care across all maternity settings. Although the primary focus remains obstetric hemorrhage, integrating selected principles from gynecologic bleeding management may further strengthen individualized and etiology-driven care.
